# Sound thoughts: How understanding the teenage brain may help us look after their ears

**DOI:** 10.3389/fnint.2022.1016842

**Published:** 2022-11-29

**Authors:** Jermy Pang, Megan Gilliver

**Affiliations:** ^1^National Acoustic Laboratories, Department of Audiological Sciences, Macquarie University, Sydney, NSW, Australia; ^2^National Acoustic Laboratories, Department of Behavioural Sciences, Macquarie University, Sydney, NSW, Australia

**Keywords:** hearing loss, hearing health, noise, adolescence, prevention, neuroscience

Neuroscience's study of brain structures and their function provides understanding of the biological underpinnings of behavior, including factors that may assist or act as barriers for programs designed to bring about behavioral change. This understanding can benefit how disease prevention and health promotion campaigns are developed and disseminated for greater effectiveness. Increasingly, public health campaigns have harnessed an understanding of neurobiology (and its complex interactions with social contexts and emotional and behavioral development of adolescents) and applied this knowledge to health promotion campaigns to enhance changed attitudes toward disease prevention and encourage healthy lifestyle choices (Bradshaw et al., 2012; Hall, 2016; Suleiman and Dahl, 2017; Pei et al., 2019).

While not an exhaustive list, some of the health focus areas that have incorporated neuroscience in their health promotion strategies include substance use (through understanding the strong association between substance use in adolescents and high levels of sensation seeking; Crawford et al., 2003), sexual health (*via* improved understanding of developing decision-making skills and the role of emotion and social influences; Ballonoff Suleiman and Brindis, 2014), and dietary health (by taking into account the neurobiological needs of safety and non-judgement; Debenham et al., 2022).

Together, these examples illustrate the importance of using our growing neuroscience knowledge about the susceptibility of risk taking, sensation seeking, and neuroplasticity in adolescence. These influences and their dynamic interplay with health conditions during this developmental phase make it an ideal time to implement positive, health-protective behavior. The consideration of neurobiological factors has been effective in increasing receptiveness to prevention messages that result in maximum engagement with young people and may be applicable across a range of health contexts and disciplines (Michie et al., 2011; Bradshaw et al., 2012; Meinke and Martin, 2017; Pei et al., 2019). One, as yet unexplored, area that may benefit from consideration is hearing health, in particular, the prevention of noise induced hearing loss (NIHL).

The impacts of hearing loss are well-documented to have far-reaching consequences that extend beyond listening and communication difficulties, impacting on personal, societal, and global levels if left untreated (Reed et al., 2019; Sheppard et al., 2020). The biological processes of how and when noise affects hearing is known—the risk of NIHL is based on the duration, frequency, and intensity of the noise exposure, regardless of the source (Clark and Bohne, 1999; Zhao et al., 2010), and so most prevention efforts are targeted at reducing the volume of sounds (i.e., reducing the risk at the source) to which people are exposed because it is more feasible to implement. Lowering volumes reduces the risk of the source (a higher target for hierarchy of control) whereas changes to duration can be limited by the nature of the activity (for example, duration of concerts, physical fitness class is less amenable to change). Most countries have regulatory requirements and governance around occupational noise exposure as part of health and safety controls. However, managing recreational noise exposure is less straightforward.

Occupational noise regulations apply regardless of the noise source (machinery or music) however, these are only directed at employees rather than attendees. Even then questions have been raised as to the effectiveness of these for music venues, and adherence by such workplaces to the regulations; (Barlow and Castilla-Sanchez, 2012; Kelly et al., 2012; World Health Organization, 2022).

For non-occupational exposures, it is difficult to develop regulations that take into account the wide range of possible high-volume recreational activities and individual variation in participation.

As a result, recreational noise exposure remains highly dependent on the choices made by individuals about what activities they participate in, for how long, and whether they choose to take any precautions to reduce the risk to their hearing.

As individual behavior remains a major determiner of the risk to hearing from noise, prevention activities aim to motivate and encourage engagement with noise-reduction. The cumulative nature of NIHL means that there is merit in focussing attention on the noise exposure behaviors of young people, with WHO estimates that more than one billion young people (aged 12–35 years) are at risk of hearing loss due to recreational exposure to loud sounds (2022). Recreational NIHL is preventable, its consequences are as detrimental comparative to occupational NIHL, and it should be made a public health priority (Murphy et al., 2018; Pienkowski, 2021), However, noise-induced hearing loss may not be detectable or treatable during adolescence given the cumulative nature of hearing damage (Williams and Carter, 2017). Thus, this opinion piece foscusses on prevention efforts that aim to reduce the risks to hearing over time.

Adolescence is a period marked by physical change and neural development and one which also encompasses identity formation and social growth that extends from 10 to 24 years (Sawyer et al., 2018). This formative time in the life course is also associated with skill learning, exploration, and risk-taking behaviors that could promote wellbeing (such as relationship building, shifts in sociocultural perspectives, and greater peer and societal engagement). Yet, adolescents may also be vulnerable to risk-taking and sensation seeking and forming negative behavioral patterns can also lead to adverse outcomes that heighten health risks (such as substance use and engaging in risky behaviors, among others) which could increase the burden of disease in later decades of life (Suleiman and Dahl, 2017; Patton et al., 2018; Pei et al., 2019). There has, therefore, been an increasing global focus on health of adolescents (currently the largest population in human history) recognizing that appropriate health investments are needed to ensure that future generations can thrive (Patton et al., 2018).

Historically, NIHL efforts focus on hearing health education and awareness building, but there is evidence to suggest that these have been limited in their effectiveness to change behavior, shift cultural norms, or improve rates of using hearing protective devices (Weichbold and Zorowka, 2007; Vogel et al., 2008; Widén, 2013; Gilles, 2014; Keppler et al., 2015; Steinberg, 2015). Prevention of noise-induced hearing loss for adolescence should aim to set up good habits to listen safely well into future when noise risks often start to increase. As the biggest risk is often from noise exposure that is specifically sought out in leisure activities, efforts aiming to reduce rather than avoid or ban noise might be most feasible. Thus, the challenge for this population is to foster or promote a positive habit to seek out sound safely that could facilitate safe sensation-seeking. Whilst it may be possible to educate adolescents to avoid extremely loud situations where acoustic shock symptoms are obvious and immediate signs of damage, much of prevention work is targeted at more subtly risky situations where damage may occur unnoticed.

Progressively, there has been greater attention on utilizing theoretical underpinnings grafted from behavior change principles to assist researchers and clinicians in better understanding hearing health behavior change. Through this, we can broaden how we conceptualize young people's attitudes, beliefs, intentions, and motivations tailor interventions that promote hearing health behavior changes (Coulson et al., 2016). But in addition to behavior change and health promotion models, our increasing understanding of neurobiology during adolescence may provide further dimensions to how to promote and foster healthy hearing behaviors. In particular, it is worth considering two significant factors associated with adolescence—sensation seeking and social influence.

Brain areas responsible for processing reward sensitivity have shown to be hyperactivated in young adults engaging in risk-taking behaviors (Telzer et al., 2014; Qu et al., 2015). This sensation-seeking appeal is further positively reinforced by peer influence. The quality of peer relationships is crucial as it can have a positive buffering effect (that serves as a protective behavior), or increase stress, thus increasing risk-taking behaviors that negatively impact health (Galván, 2013).

Such mechanisms may explain the commonly seen disconnect between knowledge of noise-exposure risks and preventative action. Despite awareness of risks, research has frequently shown individuals choosing to participate in noisy activities, and/or declining opportunities to mitigate that risk through hearing protection activities. Social norms have been implicated in young people's decisions about personal music player listening behaviors (Gilliver et al., 2012) and rejection of earplugs at music venues (Beach and Gilliver, 2019).

Sensation-seeking, too, has been noted as an important factor for music-listening. An investigation of young people (18–25 years) by Welch and Fremaux (2017) found that enjoyment of loud sounds at music venues was related to enabling positive physical and social experiences. The physical sensation of loud music heightens emotions and arousal state, masks negative emotions, and removes inhibitions. To this end, loud music and sounds promote intimacy and social identity and further emphasize the desire to adopt or adhere to social norms.

Taken together, this has implications when considering interventions that target recreational NIHL during adolescence. If the neurobiology of adolescents and young adults is dominated by social bonds, peer influence, and heightened risk-taking behaviors, then current approaches to hearing conservation campaigns that simply aim to provide knowledge and educate the harmful effects of loud noise stemming from recreational activities are incongruent with the needs of the target audience.

A transdisciplinary approach is necessary to develop health promotion programs. Researchers, public health policy decision-makers, health practitioners, and the education system are required to work collaboratively to harness the ways in which neurobiology interacts with socio-contextual factors to order to inform effective health campaigns that are meaningful and age-appropriate (Beach, 2017; Meinke et al., 2017; Beach and Gilliver, 2019). Neuroscience-informed approaches to tackling adolescent health issues should be seen as complementary to existing behavioral and more traditional approaches to disease prevention and health promotion. The premise of this innovative approach has shown to be successful at informing and educating young people, and de-stigmatizing health conditions while promoting tolerance and understanding of (neuro)biological limitations of the adolescent brain (O'Connor and Joffe, 2013).

## Summary and future directions/discussions

Adolescence is a formative period in life with a dual nature of vulnerability to risks and adaptability as an opportunity to form life-changing, health-promoting habits. With the rising number of young people at risk of recreational NIHL, there is a need for more effective ways to address this issue. The known social aspect of recreational noise experiences and the power of sensation-seeking of the adolescent brain warrants further exploration and consideration in NIHL prevention campaigns.

The application and contributions of neuroscience to inform future NIHL prevention programs for this age group will make a novel, meaningful, and innovative pursuit. It also presents an opportunity for neuroscientists who research adolescent health behaviors to explore an—as yet uncharted area of research.

Neuroscience-informed approaches to reducing recreational NIHL for young people are required to meet the needs of the developing adolescent brain. Designing age appropriate NIHL campaigns that take these factors into account may assist to increase the likelihood that interventions are efficacious and cost-effective.

## Author contributions

JP and MG contributed to the conception and subsequent preparation of this manuscript. Both authors contributed to the article and approved the submitted version.

## Conflict of interest

The authors declare that the research was conducted in the absence of any commercial or financial relationships that could be construed as a potential conflict of interest.

## Publisher's note

All claims expressed in this article are solely those of the authors and do not necessarily represent those of their affiliated organizations, or those of the publisher, the editors and the reviewers. Any product that may be evaluated in this article, or claim that may be made by its manufacturer, is not guaranteed or endorsed by the publisher.

## References

[B1] Ballonoff Suleiman A. Brindis C. D. (2014). Adolescent school-based sex education: using developmental neuroscience to guide new directions for policy and practice. Sex. Res. Soc. Policy 11, 137–152. 10.1007/s13178-014-0147-8

[B2] Barlow C. Castilla-Sanchez F. (2012). Occupational noise exposure and regulatory adherence in music venues in the United Kingdom. Noise Health 14, 86–90. 10.4103/1463-1741.95137 22517309

[B3] Beach E. F. (2017). Leisure noise and hearing. Semin. Hear. 38, 263–266. 10.1055/s-0037-1606322 29026260 PMC5634804

[B4] Beach E. F. Gilliver M. (2019). Time to listen: most regular patrons of music venues prefer lower volumes. Front. Psychol. 10, 607. 10.3389/fpsyg.2019.00607 30967814 PMC6438925

[B5] Bradshaw C. P. Goldweber A. Fishbein D. Greenberg M. T. (2012). Infusing developmental neuroscience into school-based preventive interventions: implications and future directions. J. Adolesc. Health 51(2, Supplement), S41–S47. 10.1016/j.jadohealth.2012.04.020 22794533 PMC13234207

[B6] Clark W. W. Bohne B. A. (1999). Effects of noise on hearing. J. Am. Med. Assoc. 281, 1658–1659. 10.1001/jama.281.17.1658 10235164

[B7] Coulson N. S. Ferguson M. A. Henshaw H. Heffernan E. (2016). Applying theories of health behaviour and change to hearing health research: time for a new approach. Int. J. Audiol. 55, S99–S104. 10.3109/14992027.2016.1161851 27138716

[B8] Crawford A. M. Pentz M. A. Chou C.-P. Li C. Dwyer J. H. (2003). Parallel developmental trajectories of sensation seeking and regular substance use in adolescents. Psychol. Addict. Behav. 17, 179. 10.1037/0893-164X.17.3.179 14498812

[B9] Debenham J. Newton N. Champion K. Lawler S. Lees B. Stapinski L.. (2022). Neuroscience literacy and substance use prevention: How well do young people understand their brain? Health Promot. J. 33, 395–402. 10.1002/hpja.516 34173994

[B10] Galván A. (2013). The teenage brain:sensitivity to rewards. Curr. Dir. Psychol. Sci. 22, 88–93. 10.1177/0963721413480859 PMC3992953 24761055

[B11] Gilles A. (2014). Effectiveness of a preventive campaign for noise-induced hearing damage in adolescents. Int. J. Pediatr. Otorhinolaryngol. 78, 604–609. 24507661 10.1016/j.ijporl.2014.01.009

[B12] Gilliver M. Carter L. Macoun D. Rosen J. Williams W. (2012). Music to whose ears? The effect of social norms on young people's risk perceptions of hearing damage resulting from their music listening behavior. Noise Health 14, 47–51. 10.4103/1463-1741.95131 22517303

[B13] Hall P. A. (2016). Prevention neuroscience: A new frontier for preventive medicine. Prev. Med. 86, 114–116. 26876625 10.1016/j.ypmed.2016.02.020

[B14] Kelly A. Boyd S. Henehan G. Chambers G. (2012). Occupational noise exposure of nightclub bar employees in Ireland. Noise Health 14, 148–154. 10.4103/1463-1741.99868 22918144

[B15] Keppler H. Ingeborg D. Sofie D. Bart V. (2015). The effects of a hearing education program on recreational noise exposure, attitudes and beliefs toward noise, hearing loss, and hearing protector devices in young adults. Noise Health. 17, 253–262. 10.4103/1463-1741.165028 26356367 PMC4900500

[B16] Meinke D. K. Finan D. S. Flamme G. A. Murphy W. J. Stewart M. Lankford J. E.. (2017). Prevention of noise-induced hearing loss from recreational firearms. Semin. Hear. 38, 267–281. 10.1055/s-0037-1606323 29026261 PMC5634813

[B17] Meinke D. K. Martin W. H. (2017). Development of Health Communications for Promotion of Safe Listening: A Review. Make Listening Safe, World Health Organization. Geneva: World Health Organization.

[B18] Michie S. Van Stralen M. West R. (2011). The behaviour change wheel: a new method for characterising and designing behaviour change interventions. Implement. Sci. 6, 1–12. 10.1186/1748-5908-6-42 21513547 PMC3096582

[B19] Murphy W. J. Eichwald J. Meinke D. K. Chadha S. Iskander J. (2018). CDC Grand rounds: promoting hearing health across the lifespan. Morb. Mortal. Wkly. Rep. 67, 243–246. 10.15585/mmwr.mm6708a2 29494567 PMC5861697

[B20] O'Connor C. Joffe H. (2013). How has neuroscience affected lay understandings of personhood? A review of the evidence. Public Understand. Sci. 22, 254–268. 10.1177/0963662513476812 23833053 PMC4107825

[B21] Patton G. C. Olsson C. A. Skirbekk V. Saffery R. Wlodek M. E. Azzopardi P. S.. (2018). Adolescence and the next generation. Nature 554, 458–466. 10.1038/nature25759 29469095

[B22] Pei R. U. I. Kranzler E. C. Suleiman A. B. Falk E. B. (2019). Promoting adolescent health: insights from developmental and communication neuroscience. Behav. Public Policy 3, 47–71. 10.1017/bpp.2018.30 PMC12359070 40832593

[B23] Pienkowski M. (2021). Loud music and leisure noise is a common cause of chronic hearing loss, tinnitus and hyperacusis. Int. J. Environ. Res. Public Health 18, 4236. 10.3390/ijerph18084236 33923580 PMC8073416

[B24] Qu Y. Galvan A. Fuligni A. J. Lieberman M. D. Telzer E. H. (2015). Longitudinal changes in prefrontal cortex activation underlie declines in adolescent risk taking. J. Neurosci. 35, 11308–11314. 10.1523/JNEUROSCI.1553-15.2015 26269638 PMC4532760

[B25] Reed N. S. Altan A. Deal J. A. Yeh C. Kravetz A. D. Wallhagen M.. (2019). Trends in health care costs and utilization associated with untreated hearing loss over 10 years. JAMA Otolaryngol. Head Neck Surg. 145, 27–34. 10.1001/jamaoto.2018.2875 30419131 PMC6439810

[B26] Sawyer S. M. Azzopardi P. S. Wickremarathne D. Patton G. C. (2018). The age of adolescence. Lancet Child Adolesc. Health 2, 223–228. 10.1016/S2352-4642(18)30022-1 30169257

[B27] Sheppard A. Ralli M. Gilardi A. Salvi R. (2020). Occupational noise: auditory and non-auditory consequences. Int. J. Environ. Res. Public Health 17, 8963. 10.3390/ijerph17238963 33276507 PMC7729999

[B28] Steinberg L. (2015). How to improve the health of American adolescents. Perspect. Psychol. Sci. 10, 711–715. 10.1177/1745691615598510 26581723

[B29] Suleiman A. B. Dahl R. E. (2017). Leveraging neuroscience to inform adolescent health: the need for an innovative transdisciplinary developmental science of adolescence. J. Adolesc. Health 60, 240–248. 10.1016/j.jadohealth.2016.12.010 28235453

[B30] Telzer E. H. Fuligni A. J. Lieberman M. D. Miernicki M. E. Galván A. (2014). The quality of adolescents' peer relationships modulates neural sensitivity to risk taking. Soc. Cogn. Affect. Neurosci. 10, 389–398. 10.1093/scan/nsu064 24795443 PMC4350483

[B31] Vogel I. Brug J. Hosli E. J. van der Ploeg C. P. B. Raat H. (2008). MP3 players and hearing loss: adolescents' perceptions of loud music and hearing conservation. J. Pediatr. 152, 400–404.e401. 10.1016/j.jpeds.2007.07.009 18280849

[B32] Weichbold V. Zorowka P. (2007). Can a hearing education campaign for adolescents change their music listening behavior? Int. J. Audiol. 46, 128–133. 10.1080/14992020601126849 17365066

[B33] Welch D. Fremaux G. (2017). Why do people like loud sound? A qualitative study. Int. J. Environ. Res. Public Health 14, 908. 28800097 10.3390/ijerph14080908 PMC5580611

[B34] Widén S. E. (2013). A suggested model for decision-making regarding hearing conservation: Towards a systems theory approach. Int. J. Audiol. 52, 57–64. 23088163 10.3109/14992027.2012.728724

[B35] Williams W. Carter L. (2017). Tinnitus and leisure noise. Int. J. Audiol. 56, 219–225. 10.1080/14992027.2016.1250961 27849126

[B36] World Health Organization (2022). WHO Global Standard for Safe Listening Venues and Events. Geneva: World Health Organization.

[B37] Zhao F. Manchaiah V. K. French D. Price S. M. (2010). Music exposure and hearing disorders: an overview. Int. J. Audiol. 49, 54–64. 10.3109/14992020903202520 20001447

